# The DNA Methylation Marker *ZNF671* Has Prognostic Value for Progressing Cervical Intraepithelial Neoplasia

**DOI:** 10.3390/cancers17193095

**Published:** 2025-09-23

**Authors:** Lena Dübbel, Anna Göken-Riebisch, Kristin Knoll, Juliane Hippe, Charis Marticke, Meike Schild-Suhren, Eduard Malik

**Affiliations:** 1 University Clinic of Gynaecology and Obstetrics, Carl von Ossietzky Universität Oldenburg, Ammerländer Heerstraße 114-118, 26129 Oldenburg, Germany; anna.goeken-riebisch@uol.de (A.G.-R.); charis.marticke@uol.de (C.M.); meike.schild-suhren@uol.de (M.S.-S.); eduard.malik@uol.de (E.M.); 2 Oncgnostics GmbH, Löbstedter Straße 41, 07749 Jena, Germany; kristinknoll@gmx.de (K.K.); j.hippe@epitype.de (J.H.); 3 University Clinic of Gynaecology and Obstetrics, Klinikum Oldenburg, Rahel-Straus-Straße 10, 26133 Oldenburg, Germany

**Keywords:** cervical intraepithelial neoplasia, HPV, prognostic marker, DNA methylation, *ZNF671*, GynTect^®^

## Abstract

Background/Objectives: Most cervical intraepithelial neoplasias (CINs) regress over time. Diagnostic screenings are limited and cannot identify the disease trend, which leads to the risk of overtreatment. Reliable methods are needed to preselect patients who will probably progress. The diagnostic GynTect^®^ assay offers sensitive and specific CIN identification from cervical scrapes, measuring the methylation of six marker genes. We studied the main marker (*ZNF671*) methylation on formalin-fixed paraffin-embedded (FFPE) material to determine if the kit provides prognostic information too. Methods: We tested 289 FFPE samples from 139 patients with varying CIN grades and disease trends, including regressive, persistent, progressive, and recurrent disease. Additionally, we correlated age and human papillomavirus (HPV) status with the results. Results: Although there are differences between FFPE material and cervical scrapes, we achieved a similar increase in *ZNF671* methylation with increasing neoplasia grade (dysplasia-free: 0%, CIN 1: 8.20%, CIN 2: 26.73%, CIN 3: 32.43%, carcinoma: 100%). In addition, *ZNF671* is more likely to detect recurring (27.12% of positives) and progressive (59.32% of positives) neoplasia. Patients with regressive (1.69% of positives) or persistent (11.86% of positives) trends less frequently show *ZNF671* methylation. Interestingly, patients with HPV 16 infection (52.54% of positives) and >30 years (89.83% of positives) are more likely to appear *ZNF671* methylation-positive. However, patients < 30 years with persistent neoplasia (42.86% of positives) also show methylation more frequently. Conclusions: The methylation of *ZNF671* is measurable in cervical FFPE material and has prognostic value. Since *ZNF671*-methylated samples are most likely to be progressing, we recommend the closer monitoring of patients with GynTect^®^-positive test results.

## Simple Summary

It is not possible using standard methods to predict if a precursor lesion of a cervix carcinoma will worsen or improve over time. However, only aggressive and worsening lesions need treatment with surgery. Therefore, there is a high need for markers which can give a prognosis about a disease’s trend. One possible marker for that is *ZNF671* which is also part of the GynTect^®^ kit for diagnosing cervical lesions. We compared the methylation of that marker between improving, stagnating, and worsening lesions and those which came back a second time after removal. We observed that *ZNF671* methylation is more likely in high-grade lesions and more likely in aggressive, worsening disease trends. Improving disease trends did not show marker methylation. Based on that we conclude that *ZNF671* could serve as a marker to predict disease trend and patients with *ZNF671* methylation need to be monitored closely.

## 1. Introduction

Cervix carcinoma (CxCa) is one of the most prevalent forms of cancer among the global female population [1]. Despite the implementation of regular screening procedures, including cytological analysis (Pap smear) [2] and screening for high-risk human papillomavirus (hrHPV), there has been a notable increase in incidence and mortality rates in numerous countries [1]. The rationale underpinning this approach is twofold. Firstly, the sensitivity of the Pap smear test is limited in detecting precancerous lesions [3]. Secondly, HPV screening lacks specificity due to the inability to differentiate between transient and persistent infections [4]. However, it has been demonstrated that only persistent infections are capable of inducing clinically relevant lesions [5,6,7]. Despite the high prevalence of HPV [8], the development of a lesion is observed in only 2.5–7.7% of cases [9,10]. Moreover, a high regression rate is documented, particularly among young patients [11,12]. Regrettably, there is an absence of a diagnostic method that can predict whether neoplasia will regress or progress in the future. Consequently, therapeutic conization is the prevailing standard of care when a biopsy reveals a high-grade squamous intraepithelial lesion (CIN 2/3) to avert carcinoma [13,14]. The German guideline advocates conization as a treatment option for CIN 3 cases and for cases of CIN 2 persisting for a duration exceeding 12 months. 

However, although a significant number of CIN 3 cases progress to a carcinoma, regression at this stage remains a possibility. Bruno et al. discovered a regression rate of 15.8% which exhibited a strong association with the biopsy cone interval exceeding 11 weeks [15]. Furthermore, the presence of concomitant factors such as inflammation or a CIN 3 that has been previously removed by biopsy can render the conization of CIN 3 patients superfluous. In their study, Chen et al. found a proportion of 14.1–19.4% of CIN 3 lesions that were downgraded in their postoperative pathology [16]. It has been estimated that approximately 50% of CIN 3 lesions may be overtreated [16].

Therefore, this treatment approach carries the potential for overtreatment, especially in young patients. Furthermore, surgical intervention can result in various complications, including bleeding, scarring, preterm delivery, and others [17,18]. 

To address this gap in care, there is a need for reliable methods to preselect hrHPV-positive patients with neoplasia that have a high probability of disease progression.

HPV infection has been demonstrated to induce changes in the host’s DNA methylation pattern in cervical carcinogenesis [19,20,21] and is strongly associated with neoplasia progression [22,23]. Therefore, methylation markers have been identified as a promising triage tool in the field of cervical cancer diagnostics. Presently, GynTect^®^ from oncgnostics GmbH is one of two commercially available methylation tests, in addition to the QIAsure Methylation Test (QIAGEN, Hilden, Germany). We investigated the methylation of the GynTect^®^ marker *ZNF671*, as GynTect^®^ has been demonstrated to exhibit a higher degree of specificity in comparison to QIAsure. This useful diagnostic instrument offers the sensitive and specific identification of CINs [24,25,26]. The *ZNF671* marker of the GynTect^®^ kit has been demonstrated to serve as a reliable instrument for the identification of CIN 3 and CxCa samples in cervical smears. Its performance exhibits superiority to that of HPV16/18 genotyping and PAX1 methylation alone [27]. 

Hoyer et al. tested the negative predictive value of the GynTect^®^ kit, where 67% of negative CIN 2 and 56% of negative CIN 3 showed regression in patients ≤ 29 years [28]. However, the study’s limitations include its relatively brief follow-up period and its exclusive inclusion of patients ≤ 29 years of age. Furthermore, the positive predictive value remains to be evaluated.

In recent years, a significant number of studies have centered on the methylation and subsequent downregulation of *ZNF671*. As demonstrated in the research by Zhan et al. and Zhang et al., the methylation of *ZNF671* has been shown to be a reliable predictor of a poor prognosis in various solid carcinomas, including cervical squamous cell carcinoma and endocervical adenocarcinoma [29,30]. However, these data are primarily focused on survival rates for carcinoma patients, as derived from The Cancer Genome Atlas data set. The comparisons made include the use of carcinoma to control tissue or to focus on the functional analysis of the *ZNF671* pathways.

The present study focuses on the performance and utility of *ZNF671* methylation to predict the progression of cervical precursor lesions. For this, we established the measurement of *ZNF671* methylation on FFPE material and investigated whether the methylation of *ZNF671* (implying a positive GynTect^®^ result) is more likely to occur in cases of aggressive and progressive disease trends. Furthermore, the investigation extended to determining whether this marker could provide prognostic information and serve as a potential diagnostic and prognostic tool in the future. To answer these questions, a unique patient cohort was monitored over several years in our dysplasia unit. This approach ensures the accurate classification of our samples into patients exhibiting regressing, persistent, progressive, and recurrent disease trends. To observe methylation positivity over time, two samples were included per patient. Furthermore, the impact of age and HPV status on the prognostic value was analyzed. 

Accordingly, the objective of this study is to determine the efficacy of *ZNF671* methylation as a prognostic screening tool for precursor lesions, as opposed to its use as a cancer grading tool for carcinoma, as previously established.

## 2. Materials and Methods

### 2.1. Ethics Statements

All patients were diagnosed and managed in the Department of Gynaecology and Obstetrics at Oldenburg University Hospital, and they provided written consent to use the retrieved samples and data for research purposes. This study was approved by the Medical Ethics Committee of the Carl von Ossietzky Universität of Oldenburg (ethics vote no. 2017-114, 01-2018, and 2020-187, 25 June 2021) and complies with the ethical principles for medical research outlined in the Declaration of Helsinki. This retrospective study is registered at the German Clinical Trials Register (DRKS00024987, 30 June 2021).

### 2.2. Study Population and Sample Collection

The cervical tissue was obtained from patients who visited the dysplasia unit in the Department of Gynaecology and Obstetrics at Klinikum Oldenburg University Hospital between 2016 and 2022. The cervix tissue was obtained by biopsy or conization and subsequently analyzed at the Institute of Pathology Oldenburg as FFPE (formalin-fixed, paraffin-embedded) tissue. The tissue FFPE blocks were stored at room temperature. 

In accordance with the German guideline for the prevention of cervical cancer [13], patients with CIN 1 are advised to undergo a Pap smear, HPV screening, and a colposcopy, which includes a biopsy after 12 months. Patients diagnosed with CIN 2 are advised to undergo a colposcopy, which includes a biopsy and a Pap smear, in addition to an HPV screening, at 9-month intervals. If a lesion persists for more than 24 months, has a PapIVa or higher classification, or is not fully visible, an excision procedure is recommended. The excision of a CIN 3 lesion is always performed via conization, a procedure intended to prevent the development of cervical cancer. Following a conization procedure for CIN 3 or CIN 2, patients are required to undergo postoperative care, which includes regular Pap smear tests and HPV screening at 6, 12, and 24 months, upon the outcome of the initial R0 resection. Furthermore, patients who underwent an R1 resection were required to undergo the procedure again every six months for a period of two years. In the event that a Pap smear test result is positive, or in the case of a high-risk HPV infection that persists for a period longer than two years, a colposcopy and biopsy are performed once more.

Given the screening pattern in question, patients are obliged to visit the dysplasia unit at regular intervals over the course of several years. This circumstance has enabled the establishment of clear biological groups, defined by disease trends. The inclusion criteria encompassed all patients who provided consent to participate and were treated in the dysplasia unit at least twice between the conclusion of 2016 and the onset of 2022. The inclusion of pregnant patients was deemed to be inappropriate for the purposes of this study (Table S1). The ongoing Coronavirus Disease 2019 (COVID-19) pandemic led to a decline in routine screening participation, and we were reluctant to introduce the potential bias of a SARS-CoV-2 infection. Consequently, the recruitment of new patients was halted. 

For all patients, age, biological group (follow-up check, regressive, persistent, progressive, recurrence), and HPV status (HPV 16, HPV 18, other high-risk HPV strains except 16 and 18, HPV negative) were evaluated (Table S4). The gynecologists and scientists in our workgroup have defined biological groups based on observation, biopsies, and pathology reports over the years. We decided on the biological groups of

Regressive*: patients showed a declining biopsy grade without treatment.

Persistent*: patients showed a stable biopsy grade of CIN 1 or CIN 2 without treatment.

Progressive: patients showed a worsening disease trend; these patients were treated via conization for CIN 2 or CIN 3 grade.

Recurrent patients: although dysplasia was removed already, patients showed cervical dysplasia again.

Follow-up check: dysplasia-free biopsies after CIN2 or CIN 3 removal.

*patients with CIN 3 or carcinoma biopsy needed immediate surgical treatment. Monitoring for disease trends is not possible; therefore those patients are all included in the progressive or recurrent biological group.

We tried to include two samples from two different time points (different years and CIN grades, if possible) in the disease analysis to observe the positivity of *ZNF671* over time. The mean time between the two samples per patient was 1.225 years. In the case of sample preparation failure, only one sample was included. Since we expect dysplasia-free samples to be negative for *ZNF671* methylation, we included an additional 30 follow-up check samples (dysplasia-free samples from patients after conization), which were not included in the analysis but analyzed separately to prevent the falsification of the results. 

While the FFPE material may not be ideal for triage or diagnosis, it is valuable for prognostic purposes and is the only material available for a retrospective investigation with known disease trends. 

### 2.3. DNA Isolation and Bisulfite Treatment

Two FFPE sections (5 µm) dried on a slide for 1 h at 52 °C were used from each patient sample. For dewaxing, slides were incubated 2–3 times for 5 min in xylene, followed by two times 5 min in ethanol. Dried sections were removed from the slides and lysed in a 1.5 mL tube with 280 µL DirectPCR (cell) lysis buffer (Viagen Biotech Inc., Los Angeles, CA, USA). The tissue was incubated for 20 h at 56 °C at 900 rpm, and after 19 h, 28 µL proteinase k (Carl Roth, Karlsruhe, Germany) was added. 

The lysate was bisulfite-treated with the DNA Methylation Gold Kit (Zymo Research, Irvine, CA, USA), according to the manufacturer’s alternative protocol no.1. DNA of 40 µL lysate was isolated using the same kit, according to the manufacturer’s standard protocol with 20 µL elution per sample.

### 2.4. Methylation-Specific PCR 

The *ZNF671* real-time methylation-specific PCR (qMSP) setup was performed according to an adapted protocol for the CFX Connect Real-Time PCR system (Bio-Rad Laboratories, Inc., Hercules, CA, USA): 

1. Temperature of 94 °C for 1 min.

2. Temperature of 94 °C for 15 s.

3. Temperature of 66 °C for 35 s.

4. Measurement of fluorescence.

5. Repeat step 2–4 41 times.

6. Temperature of 95 °C for 15 s.

7. Melt curve 60–95 °C, increment 0.5 °C for 5 s.

Normally, the GynTect^®^ kit measures the methylation of six marker genes (*ASTN1*, *DLX1*, *ITGA4*, *RXFP3*, *SOX17*, and *ZNF671*). However, we could not generate a signal for most markers for our samples. The marker *ZNF671* covers a product of 103 bp only, which is possible to amplify from DNA obtained from FFPE material and worked well. In addition, *ZNF671* is the most important and most meaningful marker of the GynTect^®^ kit. Therefore, we adapted the GynTect^®^ kit and used one quality control marker (*ACTB*) and only one methylation marker (*ZNF671*) instead of the complete GynTect^®^ kit. This still gives us some information about the prognostic value of the GynTect^®^ kit because it is considered positive if the total GynTect^®^ score is equal to or higher than six. A positive methylation signal for *ZNF671* is scored six in the GynTect^®^ kit. Therefore, the use of *ZNF671* methylation is reliable, but it needs to be considered that we used an adapted qRT-PCR with only one methylation and one control marker.

After qRT-PCR, the ∆Ct was calculated between the Ct value of the quality control marker *ACTB* and the Ct value of *ZNF671* (Table S4). To be positive, we need a ∆Ct ≤ 10 for *ZNF671* to *ACTB* and a Ct value for *ZNF671* < 40. These parameters are identical to the complete GynTect^®^ kit. Samples were invalid if the Ct value for the control marker, *ACTB*, did not give a signal for the respective sample or if the melt curve was shifted.

### 2.5. Statistics

#### 2.5.1. Sample Size Calculation

The sample size calculation was based on the assumption that half of the patients would exhibit progressive or aggressive disease trends, which would be indicative of *ZNF671* methylation if the hypothesis is valid. It was hypothesized that half of the patients would be observed, as patients exhibiting these disease trends are typically referred to the dysplasia unit with greater frequency. The present study was meticulously planned and formally approved by the relevant ethics committee as a monocentric pilot study, encompassing a single dysplasia unit. Concurrently, the pandemic imposed limitations on the available time for recruitment. Consequently, we opted to employ a confidence interval of 92.5% for the sample size calculation, as opposed to the conventional 95%. Furthermore, the employment of two samples per patient served to enhance the robustness of our data and methodologies. Consequently, we opted to accept the higher error rate.

We calculated the sample size with [31]

$$N={\left(\frac{1.78}{0.075}\right)}^{2}\times 0.5\times (1-0.5)=140.8$$

Therefore, we planned for 141 patients, but were able to recruit 139 patients only because 2 withdrew their consent.

#### 2.5.2. Evaluation of Test Quality Criteria 

In addition, we calculated the test quality criteria for the *ZNF671* methylation test for dysplasia grade or biological group. Sensitivity and specificity (Sens., Spec.) [32], positive and negative predictive value (PPV, NPV), prevalence, and positive and negative diagnostic likelihood ratio (DLR+, DLR−) [32] have been calculated. 

The following criteria were used for test quality criteria calculation: 

Test negative: quality criteria of qRT-PCR not met and/or no expression of the marker in qRT-PCR.

Test positive: expression of *ZNF671* and *ACTB*; Ct value within the limit of 20–40 and ∆Ct ≤ 10.

Disease positive (dysplasia grades): CIN 3, carcinoma.

Disease positive (biological groups): progressive, recurrent.

Disease negative (dysplasia grade: dysplasia-free, CIN 1, CIN 2.

Disease negative (biological groups): regressive, persistent.

#### 2.5.3. Logistic Regression

As previously delineated, the formation of equivalent groups for all the distinct categories of analysis proved unfeasible. Consequently, the data is presented in the form of percentages. However, data expressed in percentages is not typically distributed according to a normal curve. Consequently, the implementation of parametric tests is rendered infeasible. Therefore, a logistic regression was performed on the various parameters that were analyzed, and a combination of all parameters was subsequently calculated (see Table S3). The objective of this study was to determine whether patients exhibited a heightened propensity for *ZNF671* methylation in cases of more aggressive disease trends, elevated age, or distinct HPV statuses. The analyses were executed in SPSS Statistics (Version 30.0.0.0 (171), IBM GmbH, Hamburg, Germany). The various groups of the logistic regression model are evaluated using chi-square tests for significance. Model fitness is assessed by the −2 log-likelihood, the Cox & Snell R^2^, and the Nagelkerkes R^2^.

## 3. Results

### 3.1. Transfer of the GynTect^®^ Kit to Cervical FFPE Tissue

**Figure 1:**
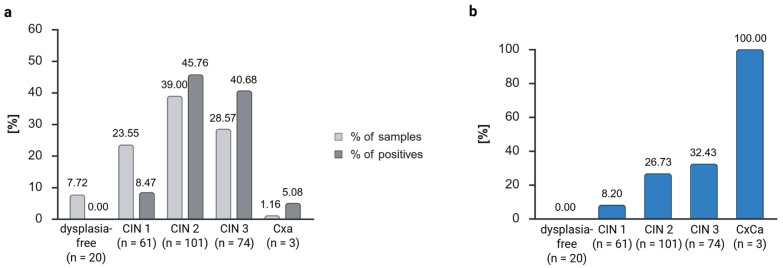
Sample distribution and distribution of positives with *ZNF671* methylation among the dysplasia grades (**a**). Amount of *ZNF671* methylation positivity for the different dysplasia grades (**b**). n = 259 samples from 139 patients. CIN, cervical intraepithelial neoplasia; CxCa, cervix carcinoma. Created in BioRender. Dübbel, L. (2025) https://BioRender.com/z5hps7f, accessed on 22 September 2025.

Due to the variation in sample numbers, the percentages of positive and negative samples were calculated (Figure 1a). The actual numbers of samples are included in Table 1, which shows the descriptive statistics. In order to avert the fabrication of results, the samples from the dysplasia-free follow-up checks (n = 30) were excluded from the analysis. With that, 259 samples were analyzed. The diagnosis of CIN 3 necessitates the surgical excision via the conization of the dysplasia. Consequently, carcinomas are sporadic in our dysplasia unit, and we were only able to include three samples (1.16%) in our study. Dysplasia-free samples constituted 7.72% of the total, with the majority (16 of 20) belonging to the category of regressive dysplasia. The majority of patients exhibited dysplasia of higher grades. Therefore, 61 samples with CIN 1 (23.55%), 101 samples with CIN 2 (39.00%), and 74 samples with CIN 3 (28.57%) were included (Figure 1a, Table 1).

We proceeded to assess the methylation status of the marker gene *ZNF671* in patients with dysplasia-free, CIN 1, CIN 2, CIN 3, or cervical carcinoma samples (Figure 1b). It was observed that all samples that were free of dysplasia did not exhibit *ZNF671* methylation and were negative (see Figure 1b). As the severity of dysplasia increased, there was an observed increase in the positivity of *ZNF671* methylation, ranging from 8.20% in CIN 1, 26.73% in CIN 2, 32.43% in CIN 3, to 100% in carcinoma. In relation to the totality of positive cases, 8.47% of the positive samples were classified as CIN 1, 45.76% were designated as CIN 2, 40.68% were categorized as CIN 3, and 5.08% were diagnosed as carcinomas (Figure 1a).

Consequently, an increase in positivity could be reproduced, albeit with lower positivity rates. This phenomenon can be attributed to the fixed and degraded biological material, in contrast to the cervical scrapes that are conventionally utilized.

### 3.2. The Prognostic Value of ZNF671 Corresponds to Severe Disease Trends 

**Figure 2:**
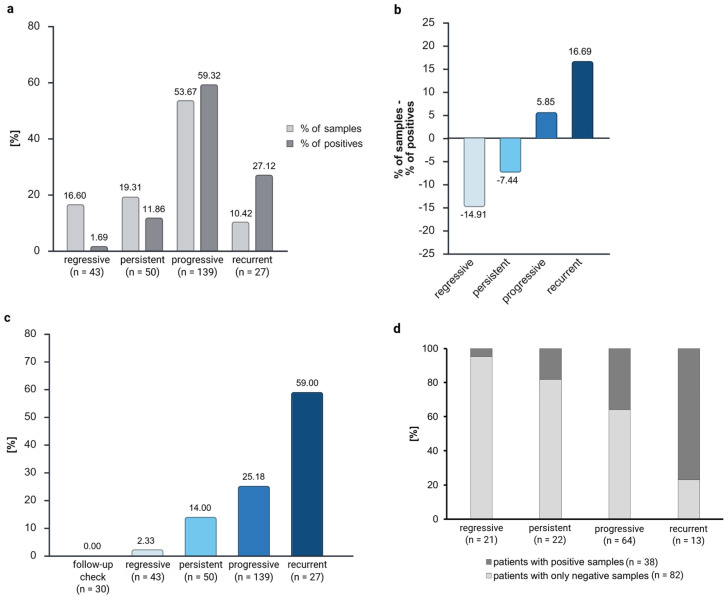
Sample distribution and *ZNF671* methylation positivity distribution among the biological groups (**a**). The difference between the distribution of the number of samples and *ZNF671* methylation positivity distribution is clarified in (**b**) for better visualization. Amount of *ZNF671* methylation positivity for the biological groups (**c**). (**a**–**c**) shows the analysis of all 289 samples, while (**d**) shows the analysis of patients with positive and only negative samples. Created in BioRender. Dübbel, L. (2025) https://BioRender.com/19o76vu, accessed on 22 September 2025.

We compared the methylation levels across the biological groups to investigate the prognostic value of *ZNF671* from the GynTect^®^ kit (Figure 2). As previously stated, the samples of follow-up checks (n = 30, 10.38%) were excluded from the analysis. Consequently, the remaining samples were classified into the following disease trends: regressive (n = 43, 16.60%), persistent (n = 50, 19.31%), progressive (n = 139, 53.67%), and recurrent (n = 27, 10.42%) (Table 1). A comprehensive evaluation of all dysplasia-free samples from subsequent follow-up checks yielded negative results. Of the 59 *ZNF671* methylation-positive samples, 1 was regressing (1.69%), 7 were persistent (11.86%), 35 were progressive (59.32%), and 16 showed recurrent disease trends (27.12%) (Figure 2a).

Therefore, regressive samples exhibited 14.91% less positivity than their sample number, persistent samples demonstrated 7.44% less positivity than the sample amount, progressive samples exhibited 5.65% higher positivity than the sample amount, and recurrent samples exhibited 16.69% more positivity than the sample amount (Figure 2b).

A comparison of the biological groups revealed that 0% of the follow-up checks, 2.33% (1 of 43) of the regressive samples, 14.00% (7 of 50) of the persistent samples, 25.18% (35 of 139) of the progressive samples, and 59.00% (16 of 27) of the recurrent samples were *ZNF671* methylation-positive (Figure 2c). Therefore, the positivity of *ZNF671* methylation demonstrated a correlation with the aggressiveness of the disease trend (Figure 2c). Except for one outlier, no samples that demonstrated regression exhibited *ZNF671* methylation.

This effect can be observed in the context of logistic regression as well (Table S3). Despite the suboptimal fit of the model (indicated by the −2 log-likelihood: 243.370, Cox&Snell R^2^: 0.156, and Nagelkerkes R^2^: 0.246), a noteworthy proportion of the sample methylation can be predicted with a high degree of accuracy (81.3 %, see Table S3). It has been determined that a methylated sample is recurrent with a probability of 62.5 times the probability of a regressive outcome (*p* = 0, Exp(B) = 0.016), 8.9 times the probability of a persistent outcome (*p* = 0, Exp(B) = 0.112), and 4.33 times the probability of a progressive outcome (*p* = 0.001, Exp(B) = 0.231).

This result was consistent across both the analysis of samples (Figure 2a–c) and the analysis of patients (Figure 2d). 

An analysis was conducted on two samples per patient, if available, to ascertain whether the patient exhibited consistent positive or negative results. Among the 139 patients, 82 exhibited negative results in both samples. Twenty samples exhibited a mixture of positive and negative results, while eighteen samples yielded positive outcomes in both tests. For 19 patients, the analysis of a second sample was not feasible.

With more aggressive disease trends, the number of patients with samples that were only negative for *ZNF671* methylation decreased. We found that 95.24% of patients with a regressive diagnosis had both samples negative for *ZNF671*-methylation. For persistent disease trends, it was 81.82 % of patients, 64.06% for patients with progressive disease trends, and 23.08% for patients with recurrent disease trends, which showed only *ZNF671* methylation-negative samples.

This finding indicates that the methylation process occurs over time or in cases of higher dysplasia grade.

**Figure 3:**
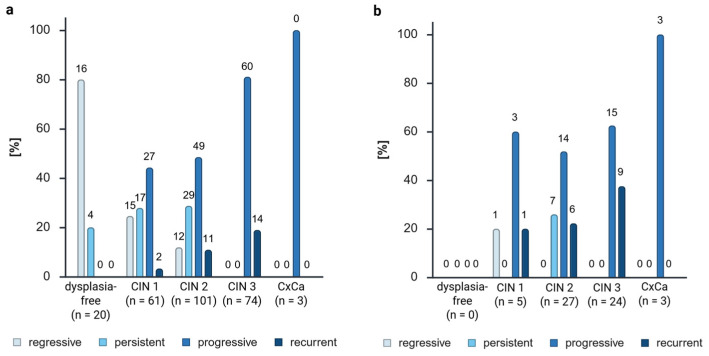
Sample distribution (**a**) and *ZNF671* methylation positivity distribution (**b**) in a combined analysis of dysplasia levels and biological groups. Absolute numbers (n) are shown above the bars. CIN, cervical intraepithelial neoplasia. Created in BioRender. Dübbel, L. (2025) https://BioRender.com/0kq80y4, accessed on 22 September 2025.

The combination of biological groups (Table S2) and grades exhibited a higher proportion of aggressive (progressive and recurrent) samples with a higher CIN grade (progressive: 0% of dysplasia-free patients, 44.26% of CIN 1, 48.51% of CIN 2, 81.08% of CIN 3, and 100% of carcinoma; recurrent: 0% of dysplasia-free patients, 3.28% of CIN 1, 10.89% of CIN 2, 18.92% of CIN 3, and 0% of carcinoma) (Figure 3a). Furthermore, a lower number of regressive samples (80% of dysplasia-free patients, 24.59% of CIN 1, 11.88% of CIN 2, 0% of CIN 3, and 0% of carcinoma) and persistent samples (20% of dysplasia-free patients, 27.87% of CIN 1, 28.71% of CIN 2, 0% of CIN 3, and 0% of carcinoma) with a higher CIN grade (Figure 3a) could be observed. However, given the standard procedure of the conization of CIN 3 cases, it is not possible to observe the regression or persistence of a CIN 3.

As illustrated in Figure 3b, the distribution of positive samples corroborated the observations documented in Figure 2. The majority of methylated samples exhibited an aggressive phenotype (progressive + recurrent = 84.18% of positives), which demonstrated the highest number of methylated samples across all CIN grades (progressive: 60% of CIN 1 positives, 51.85% of CIN 2 positives, 62.50% of CIN 3 positives, and 100% of carcinoma positives; recurrent: 20% of CIN 1 positives, 22.22% of CIN 2 positives, 37.50% of CIN 3 positives, and 0% of carcinoma positives).

Notably, one methylated regressive CIN 1 sample (20% of CIN 1 positives) and seven persistent CIN 2 samples (25.93% of CIN 2 positives) were observed. 

Furthermore, we calculated for the sensitivity, specificity, positive predictive value (PPV), negative predictive value (NPV), prevalence, positive diagnostic likelihood ratio (DLR+), and negative diagnostic likelihood ratio (DLR−) for the *ZNF671* methylation test. This calculation was performed to determine the diagnostic accuracy of the test depending on dysplasia grades and biological groups. Given the compromised integrity of the DNA present in our FFPE material, it was anticipated that the sensitivity would be diminished, a phenomenon which is evident in both calculations (Table 2). Conversely, the specificity is high for dysplasia grades (82.42%), particularly for the biological groups (91.40%), thereby substantiating our conclusions derived from Figure 2 and Figure 3 When utilizing solely progressive and recurrent samples as disease-positive instances in the calculations, the positive predictive value (PPV) is elevated, reaching 86.44%. However, the negative predictive value (NPV) is diminished, attaining 42.50%, due to the presence of numerous positive persistent samples. Additionally, the DLR+ and − are found to be inadequate for both calculations. The reliability of these results is contingent upon the reference system employed for disease-positive samples. A critical discussion point is whether persistent samples or CIN 2 samples should be incorporated into the calculations. Therefore, the optimal approach for determining the prognostic value of the *ZNF671* methylation test is not yet apparent. Nonetheless, the inclusion of these data was deemed necessary to substantiate the elevated specificity of the assay and to demonstrate that enhanced diagnostic precision is attained through the utilization of biological groups as opposed to the exclusive reliance on CIN grades.

### 3.3. The Role of HPV Infections in Cervical Dysplasia

**Figure 4:**
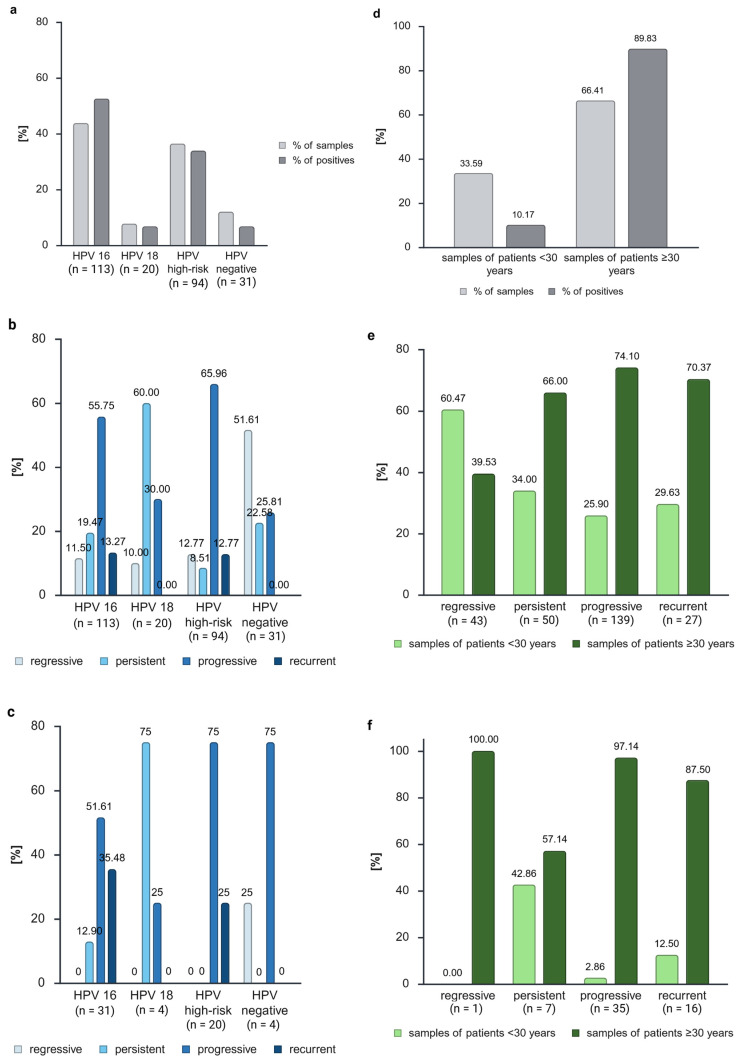
Analysis of sample distribution and *ZNF671* methylation positivity distribution by HPV status (**a**) and HPV status combined with biological groups (**b**,**c**). Analysis of sample distribution and *ZNF671* methylation positivity distribution by the age categories < 30 years and ≥30 years (**d**) and age combined with biological groups (**e**,**f**). Created in BioRender. Dübbel, L. (2025) https://BioRender.com/r5jz84m, accessed on 22 September 2025.

The samples included in this study were either infected with HPV 16 (43.75%), HPV 18 (6.94%), other high-risk HPV types (38.54%), or were uninfected (10.76%) (Figure 4a, Table 1). A higher proportion of *ZNF671* methylation-positive samples was observed in HPV 16-infected samples (52.54%) compared with the total sample size (43.80%). For high-risk HPV and negative samples, the reverse outcome was observed, exhibiting a lower number of positives compared with the sample volume. The distribution of these results into the various biological groups (Figure 4b, Table S2) demonstrated that the majority of HPV 18-positive samples were persistent samples (n = 12, 60%), and three of these were methylation-positive (Figure 4c). The majority of HPV 16-positive samples exhibited progressive characteristics (n = 63, 55.75% of the samples, 51.61% of the positives). However, a notable increase in positivity was observed among recurrent samples (13.27% of the samples, 35.48% of the positives). The majority of the samples exhibiting regression were HPV-negative, accounting for 51.61% of the total. For samples infected with other high-risk strains, progressive and recurrent samples exhibited a comparable increase in positivity, proportionate to their sample size (progressive: 65.96% of samples, 75% of positives; recurrent: 12.77% of samples, 25% of positives). Notably, the sole *ZNF671* methylation-positive regressive sample was HPV-negative. The remaining GynTect^®^-positive and HPV-negative samples exhibited a progressive pathology (n = 3).

As anticipated, the findings of the logistic regression analysis revealed no statistical significance (Table S3). We defined HPV 16 as the reference category and obtained a model fitting with a −2 log-likelihood of 289.167, Cox&Snell R^2^ of 0.010, and Nagelkerkes R^2^ of 0.016. The regression coefficients were −0.790 for HPV negative (*p* = 0.169), −0.395 for high-risk HPV (*p* = 0.220), and -0.266 for HPV 18 (*p* = 0.655).

### 3.4. The Influence of Age on Cervical Dysplasias

A significant proportion of the patient population in the dysplasia unit was aged 30 years or older, constituting approximately two-thirds of the total cases (Table 1). These patients exhibited a higher propensity for *ZNF671* methylation, suggesting that 89.83% of their samples were methylated (Figure 4d). A subsequent analysis of the distribution according to age and biological groups (see Figure 4e, Table S2) revealed that patients exhibiting regressive disease trends were predominantly under the age of 30 (n = 26, 60.47%). Meanwhile, 70.37% of the recurrent trends and 74.10% of the progressive trends were observed among patients with a minimum age of 30 years. A surprising finding was that only 1 of the 35 methylated progressing samples (2.86%) and 2 of the 16 methylated recurrent samples (12.50%) were from patients younger than 30 years. However, these groups constitute 25.9% (36 of 139) and 29.63% (8 of 27) of the samples in these biological groups (Figure 4e,f).

Conversely, a higher positivity rate has been observed in persistent patients under 30 years of age. A total of 34.00% (17 of 50) of the persistent samples were from patients younger than 30 years, yet these patients constituted 42.86% (3 of 7) of the positive cases within this biological group (see Figure 4f).

The sole *ZNF671*-positive regressive sample was obtained from a patient who was at least 30 years of age.

To check for a potential correlation between age and *ZNF671* methylation, we completed a logistic regression analysis (Table S3) with age as the independent variable. However, the resultant *p*-value (*p* = 0.738) did not demonstrate statistical significance. The regression coefficient was determined to be 0.005 and the model fit was 79.6%. The −2 log-likelihood was found to be 292.418, the Cox&Snell R^2^ was 0, and the Nagelkerkes R^2^ was 0.001. Therefore, the changes in *ZNF671* methylation observed cannot be attributed to methylation changes resulting from the aging process; instead, they are more likely to be attributable to carcinogenesis.

## 4. Discussion

The primary marker, *ZNF671*, was transferred from the GynTect^®^ kit, which is used for cervical scrapes, to FFPE material due to the unavailability of cervical scrapes from different grades within the disease analysis. In order to investigate the hypothesis that *ZNF671* methylation is more likely in aggressive and progressive disease trends, only FFPE material was accessible. The transfer of the entire kit proved unfeasible due to the suboptimal performance of five of the six markers, which likely resulted from the amplicons’ inadequate length for the fragmented DNA. The utilization of primers with a gene product length of less than 100 base pairs has been demonstrated to be a viable solution to this challenge. Nevertheless, it was determined that adherence to the original primers was necessary to ensure the maintenance of comparability.

Notwithstanding, the kit transfer proved successful, as evidenced by the reliability of *ZNF671* as a marker for GynTect^®^ positivity. The majority of samples positive for this marker are also GynTect-positive [33] and the positivity of *ZNF671* is indicative for the positivity of the GynTect^®^ kit, given its high score.

Another factor that must be considered is the size of the patient cohort. Patients with high-grade dysplasia are more frequently referred for consultation, which precluded the possibility of creating groups of patients of equivalent sizes. Consequently, a higher proportion of patients with CIN 2 (n = 101) and CIN 3 (n = 74) were included, while patients who were dysplasia-free (n = 50), CIN 1 (n = 61), and especially carcinoma (n = 3) were underrepresented. A diagnosis of CIN 3 necessitates the procedure of conization to prevent carcinoma development. Consequently, the inclusion of additional samples from patients diagnosed with cervical carcinoma was not feasible. This imbalanced distribution of patient groups results in data analysis using percentages rather than absolute numbers.

The reliability of *ZNF671* methylation was confirmed through the observation that the proportion of methylated (positive) samples increased with increasing dysplasia grades, reaching 100% positivity in cases of cervical carcinoma. This finding aligns with the results reported in previous studies using the GynTect^®^ kit. 

Schmitz et al. found positivity of the GynTect^®^ kit of 1.5% in dysplasia-free samples, 20% in CIN 1, 44.4% in CIN 2, 61.2% in CIN 3, and 100% in carcinoma samples in cervical scrapes [25]. In contrast, *ZNF671* positivity in hrHPV-positive patients has been documented to range from 10% in dysplasia-free cases to 10.5% in CIN 1, 36% in CIN 2, 67% in CIN 3, and 89% in carcinoma [34]. However, it should be noted that these findings were obtained under different qRT-PCR conditions than those employed in our study.

We observed lower positivity rates, which can be explained by the degradation of the biological material. This also explains the low sensitivity of the *ZNF671* methylation of 35.06%, if we use the dysplasia grades for calculation, in our study. While 28.57% of the samples were classified as CIN 3, these samples constituted 40.68% of all positive samples. Overall, 39.00% were classified as CIN 2 specimens, yet these specimens constituted 45.76% of all positive cases. In comparison, 23.55% of all samples were classified as CIN 1, yet these samples constituted a mere 8.47% of the positive cases. As anticipated, dysplasia-free samples yielded negative results, thereby substantiating the specificity of the *ZNF671* marker for FFPE material. This can also be observed in the calculation if we define CIN 3 and CxCa as disease positive, which gives a specificity of 82.42%. However, to mitigate the risk of data fabrication, the dysplasia-free follow-up check samples were excluded from analysis. Only dysplasia-free samples from regressive and persistent disease trends (n = 20) were included in the analysis. 

In order to ascertain the prognostic value of *ZNF671*, a division of the samples into distinct biological groups was conducted, including follow-up check (not included in the percental analysis) and regressive, persistent, progressive, and recurrent disease trends. The initial hypothesis of this study was that there would be a predominance of positivity in more aggressive disease trends and that *ZNF671* would not be methylated in regressive samples.

The delta of the percentage of positives to the percentage of samples was then analyzed. A subsequent comparison of the *ZNF671* positivity to the sample amount revealed the following percentages: −14.91% for recurrent samples, −7.44% for persistent samples, 5.65% for progressive samples, and 16.69% for recurrent samples. This finding indicated a higher *ZNF671* positivity rate with increasing disease aggression. However, it was observed that only 27.12% of the positive cases were from recurrent samples, while 59.32% were from progressive samples due to the disparity in the patient cohort. The analysis of the number of positives within the biological group corroborates this finding (59% of recurrent samples, 25.18% of progressive samples, 14.00% of persistent samples, 2.33% of regressive samples, and 0% of follow-up controls were *ZNF671* methylation-positive). The results of the analysis were evident in both the examination of the samples and the patients themselves. A collective observation indicated an escalating degree of methylation in accordance with the severity of the disease’s progression.

The calculation of the diagnostic accuracy with the progressive and recurrent biological groups as disease positive showed, as expected, a low sensitivity of 30.72% and a high specificity of 91.40%. The positive predictive value of 86.44% showed that progressive and recurrent samples have a high probability of showing *ZNF671* methylation. Despite the suboptimal diagnostic accuracy of FFPE material, necessitating repeated testing on cervical smears, the findings suggest that *ZNF671* methylation may serve as a specific prognostic marker for cervical lesions.

This finding is also supported by the logistic regression with *ZNF671* methylation as the dependent variable and disease trend as the independent variable (Table S3). The model showed 81.3% correct prediction, with a significantly higher probability that methylated samples are recurrent than other disease trends. 

These findings corroborate our primary hypothesis and underscore the prognostic significance of *ZNF671* methylation.

As anticipated, biological groups exhibiting a more aggressive profile, characterized by progressive and recurrent characteristics, demonstrated higher grades of dysplasia. This finding aligns with the prevailing knowledge [35] that higher CIN grades are less prone to regression. A higher grade of dysplasia was associated with a higher number of progressive samples.

While the majority of the progressive and recurrent samples exhibited methylation, one regressive CIN 1 sample and seven persistent CIN 2 samples were also methylation-positive. The presence of a single regressive methylated CIN 1 sample may be indicative of an outlier or a false positive result. Hoyer et al. [28] hypothesized that more than 90% of regressive samples are GynTect^®^-negative. Their hypothesis cannot be empirically validated with the FFPE tissue utilized in this study due to the presence of a higher percentage of negative samples from all biological groups. This phenomenon can be attributed to DNA degradation and low DNA concentrations, resulting in elevated Ct values and a low test sensitivity. However, a significant proportion of the regressive samples (97.67%) were negative for *ZNF671* methylation, thereby corroborating the hypothesis proposed by Hoyer et al. [28]. Chen et al. also found that negativity for *ZNF671* methylation could predict the regression of CIN 3 lesions [16]. They concluded that *ZNF671* methylation should be used in combination with cytology and HPV screening to improve the predictive performance further, with the aim of enabling the individualized management of CIN 3 treatments in the future. 

Moreover, 86.44% of the positive samples exhibited aggressive disease trends (progressive and recurrent), thereby substantiating our hypothesis regarding the prognostic value of the GynTect^®^ kit. 

The persistent biological group constituted 11.86% of the positives (7 positives of 59 samples, all CIN 2). This prompts further inquiry into the temporal progression of these patients, particularly whether they are the ones who will eventually manifest an aggressive dysplasia. Consequently, the close monitoring of these patients is strongly recommended, and we suggest using the GynTect^®^ kit in combination with Pap smear and HPV screening, if our results can be confirmed on cervical smears. Regrettably, only one of the patients in question underwent a subsequent visit to our dysplasia unit (after a period of one year), during which time no dysplasia was identified. A future study should concentrate on that aspect. 

Nevertheless, if we specifically compare the biological groups of CIN 2 (because for CIN3+, no regressive or persistent samples can be observed), we can see aggressive disease trends (progressive + recurrent) for 54.06% of the GynTect^®^-negative samples and 16.22% regressive disease trends. Meanwhile for GynTect^®^-positive CIN 2 samples, we see 74.07% aggressive disease trends and no regressive ones. With these results, we can see that progressing dysplasia can show *ZNF671* methylation already in low dysplasia grades, which supports the prognostic value of the GynTect^®^ kit and demonstrates that this marker is not only a marker for cancer grading or CIN 3+ lesions, but could be used for lower dysplasia grades as well. Generally, CIN 2 cases demonstrate 32% persistency, 22% progression, and 40–58% regression [36,37]. However, in this case, the observed values significantly exceed these benchmarks, which is attributable to the fact that more aggressive and persistent disease trends are referred to dysplasia units.

Taken together, if it could be confirmed on cervical smears that samples showing *ZNF671* methylation already, as a CIN 1, CIN 2, or CIN 3, are the ones that will likely progress to a carcinoma in the future, these lesions should be removed at the earliest possible stage. The extent of the conization, i.e., the degree of the surgical margin, has been demonstrated to correlate with the occurrence of adverse effects. This is of particular pertinence for patients who intend to conceive in the future, as various side effects associated with conizations have the potential to adversely impact fertility and birth [38,39,40]. Conversely, if the findings from this study and those of Chen et al. [16] can be validated, indicating that negative *ZNF671* methylation suggests the regression of the lesion, a watchful waiting approach for these lesions, accompanied by annual testing via Pap smear, HPV screening, and *ZNF671* methylation, should be considered to avert overtreatment. 

The analysis of HPV status showed a higher number of positives for HPV 16-infected samples and a lower number of positives for other high-risk HPV-infected and uninfected samples. HPV 18 infection showed similar amounts of samples and positives. HPV 18 infection demonstrated comparable proportions of samples and positives. Hoyer et al. observed analogous results, with a higher positivity for samples with HPV16/18 infection and less methylation in samples with other high-risk HPV types [28]. A thorough examination of the distribution of HPV statuses within the samples revealed that the majority of samples infected with HPV 18 exhibited persistent characteristics, and three of these samples demonstrated *ZNF671* methylation. This outcome is notable given the prevailing consensus that HPV 18 is classified as a high-risk strain, with a strong propensity to lead to disease progression. However, the potential progression of these samples remains uncertain. As anticipated, the majority of HPV-negative samples exhibited a regressive pattern. For the remaining HPV status groups, progressive samples were most prevalent, and only progressive and recurrent samples exhibited *ZNF671* methylation.

The methylated regressive CIN 1 outlier was also HPV-negative and exhibited a remarkably elevated *ZNF671* Ct value of 38.93 (Figure S1A, Table S4), thereby substantiating the hypothesis that this is a false positive due to the late PCR amplification, as this combination is improbable. This prompts the question of whether a lower cut-off at a given Ct value would offer a higher prognostic value. A decrease in Ct values was observed in correlation with an increase in dysplasia grade and the biological group’s aggressiveness. Therefore, it can be posited that a cut-off at a Ct value of, for example, 38 would enhance the kit’s prognostic performance. To evaluate this, it would be necessary to repeat these experiments with cervical scrapes from known biological groups. Another possibility is the adjustment of the ∆Ct values or the implementation of a cut-off for the Ct value of *ACTB*.

In the context of cervical dysplasia and carcinoma, a common practice is to categorize samples according to the age of the patients, distinguishing between those under and over 30 years of age. This approach is rooted in the understanding that immunologic pressures vary significantly across different age demographics. Our patient cohort had a mean age of 35.13 years. It is noteworthy that approximately three-quarters of the samples obtained from patients in progressive and recurrent biological groups were from individuals aged 30 years and older. Nevertheless, the logistic regression showed that *ZNF671* methylation is not correlated with age, but with the disease trend (Table S3). Therefore, the differences in *ZNF671* methylation are not due to aging, but more likely from the carcinogenesis process. 

The majority of regressive samples were collected from patients under 30 years of age (60.47%). However, a mere 12.50% of methylated recurrent samples were derived from patients under the age of 30. For methylated progressive samples, 2.86% were from patients under 30 years of age, and 97.14% were from patients over 30 years of age. Given the temporal limitations of our observation period and the clinical guideline of the conization of CIN 3 cases and recurrences, we are unable to provide a definitive answer to the question regarding the rationale behind the high prevalence of progressive and recurrent patients with a negative GynTect^®^ result. One potential explanation for this phenomenon is that the immune system plays a regulatory role in controlling the progression of dysplasia, thereby limiting further development. As previously indicated, FFPE material is not appropriate for the discussion of negative predictive value. 

Despite comprising only 34.00% of the sample (n = 17), patients under 30 years of age accounted for 42.86% of the positives (n = 3). This prompts further inquiry into the potential progression of these young patients within the coming years, thereby substantiating our hypothesis regarding the prognostic value of the GynTect^®^ kit. Consequently, close monitoring is imperative to observe emerging disease trends.

## 5. Conclusions

We conclude that the transfer of the primary marker gene *ZNF671* of the GynTect^®^ kit to FFPE material was successful, yielding reliable results. Our research has validated the hypothesis that *ZNF671* methylation has prognostic value for cervical samples. The positivity of the marker was found to be higher in cases with a worse prognosis and disease progression. The analysis revealed that samples exhibiting no further therapeutic need were non-methylated, with the exception of a single outlier. The vast majority of methylated samples exhibited aggressive disease trends, necessitating treatment to avert the development of carcinoma. Nevertheless, these findings first need to be confirmed in cervical smears with the complete GynTect^®^ kit.

The methylated samples of CIN 1 and 2 are associated with aggressive progression and, as such, necessitate early treatment. Furthermore, it is hypothesized that GynTect^®^-positive persistent samples are more likely to progress in the future and should be monitored more closely and also be treated as early as possible when the side effects of conization are smaller. This consideration assumes particular significance for patients who aspire to conceive in the future. Conversely, a CIN 2 with a negative GynTect^®^ result is more likely to persist or regress. Consequently, it may be adequate to merely monitor the dysplasia rather than undertaking surgical intervention. This approach might be considered acceptable for affected patients as they undergo watchful waiting with a negative cancer marker test result. If our findings can be confirmed in cervical smears, we suggest that the GynTect^®^ kit with its main marker *ZNF671* should be integrated into routine screening in addition to Pap smear and HPV screening to gain preliminary insights into the probability of dysplasia progression. Its application in dysplasia units as an ancillary information source has the potential to reduce the risk of overtreatment. Conversely, it could facilitate earlier, and therefore less extensive, treatment for lesions with a high probability of progression.

## Figures and Tables

**Table 1:** Descriptive statistics of samples with the total *ZNF671* methylation-negative and -positive samples. “% positive” describes the positivity rate within the biological group (*ZNF671* methylation positives/(total/100)), “% of samples” describes the percentage of samples, compared to all samples, in that biological group (total/(259/100), and “% of positives” describes the percentage of positives, compared to all positives, in that biological group (*ZNF671* methylation positives/(59/100)).

	Total [n]	*ZNF671* Methylation-Negative [n]	*ZNF671* Methylation-Positive [n]	% Positive	% of Samples	% of Positives
dysplasia-free	20	20	0	0	7.72	0
CIN 1	61	56	5	8.20	23.55	8.47
CIN 2	101	74	27	26.73	39.00	45.76
CIN 3	74	50	24	32.43	28.57	40.68
carcinoma	3	0	3	100	1.16	5.08
follow-up check	0	0	0	0	0	0
regressive	43	42	1	2.33	16.60	1.69
persistent	50	43	7	14.00	19.31	11.86
progressive	139	104	35	25.18	53.67	59.32
recurrent	27	11	16	59	10.42	27.12
HPV 16	113	82	31	27.43	43.80	52.54
HPV 18	20	16	4	20.00	7.75	6.78
high-risk HPV	94	74	20	21.28	36.43	33.90
HPV-negative	31	27	4	12.90	12.02	6.78
samples of patients < 30 years	87	81	6	6.90	33.59	10.17
samples of patients ≥ 30 years	172	119	53	30.81	66.41	89.83

**Table 2:** Quality criteria for the *ZNF671* methylation test. For the calculation of diagnostic accuracy regarding dysplasia grades, CIN 3 and carcinoma samples are rated as “disease positive”. For the calculation of diagnostic accuracy regarding biological groups, progressive and recurrent samples are rated as “disease positive”. CIN, cervical intraepithelial neoplasia; DLR+, positive diagnostic likelihood ratio; DLR−, negative diagnostic likelihood ratio; NPV, negative predictive value; PPV, positive predictive value; Prev, prevalence; Sens, sensitivity; Spec, specificity.

		Sens	Spec	PPV	NPV	Prev	DLR+	DLR−
dysplasia grade	%	35.06%	82.42%	45.76%	75.00%	29.70%	1.994	0.788
	95%-width of CI	24.53–46.78%	76.1–87.65%	32.72–59.25%	68.4–80.84%	24.2–35.7%	1.288–3.089	0.66–0.941
biological groups	%	30.72%	91.40%	86.44%	42.50%	64.10%	3.572	0.758
	95%-width of CI	23.81–38.34%	83.75–96.21%	75.02–93.96%	35.56–49.67%	57.9–69.9%	1.772– 7.198	0.673–0.854

## Data Availability

The original contributions presented in this study are included in the article/Supplementary Material. Further inquiries can be directed to the corresponding author.

## Supplementary Materials

The following supporting information can be downloaded at: https://www.mdpi.com/article/10.3390/cancers17193095/s1, Figure S1. Boxplot of the ZNF671 qRT-PCR Ct value distribution over the biological groups (A), age categories (B), dysplasia status (C), and HPV status (D). Ct, cycle threshold; HPV, human papilloma virus; qRT-PCR, quantitative real-time polymerase chain reaction. Table S1. Depiction of a priori defined inclusion and exclusion criteria. CIN, cervical intraepithelial neoplasia. Table S2. Descriptive statistics of combinational analysis of dysplasia grade to biological groups (a), HPV status to biological groups (b), and biological groups to age (c), each with analysis of all samples and only positive samples. Follow-up samples are not included. CIN, cervical intraepithelial neoplasia; HPV, human papillomavirus. Table S3. SPSS output of the logistic regression, analyzing the disease trends (a), the HPV status (b), the age (c), the dysplasia grades (d), and all parameters (e). Table S4. Summary of the anonymous patient data, with patient number, age at sampling, CIN grade, biological group, HPV status, GynTect result, Ct value of ZNF671, Ct value of ACTB, and the ∆Ct value of Ct ACTB-Ct ZNF671. CIN, cervical intraepithelial neoplasia; Ct, cycle threshold; no, number.

## Abbreviations

The following abbreviations are used in this manuscript:

bp	basepair(s)
°C	degrees Celsius
CI	confidence interval
CIN	cervical intraepithelial neoplasia
Ct	cycle threshold
CxCa	cervix carcinoma
DLR+	positive diagnostic likelihood ratio
DLR−	negative diagnostic likelihood ratio
DNA	deoxyribonucleic acid
DEPC	Diethylpyrocarbonate
FFPE	formalin-fixed paraffin-embedded
h	hour(s)
HPV	human papilloma virus
hr	high-risk
HSIL	high-grade squamous intraepithelial lesion
l	liter
LSIL	low-grade squamous intraepithelial lesion
m	meter
min	minute
NPV	negative predictive value
PPV	positive predictive value
Prev	prevalence
qRT-PCR	quantitative real-time polymerase chain reaction
rpm	revolution per minute
RT	room temperature
s	second(s)
Sens	sensitivity
Spec	specificity
