# Supplementary figures and images for: The DNA Methylation Marker ZNF671 Has Prognostic Value for Progressing Cervical Intraepithelial Neoplasia
Source: Cancers (Basel). 2025 Sep 23;17(19):3095. doi: 10.3390/cancers17193095 (PMC12524222; doi:10.3390/cancers17193095)

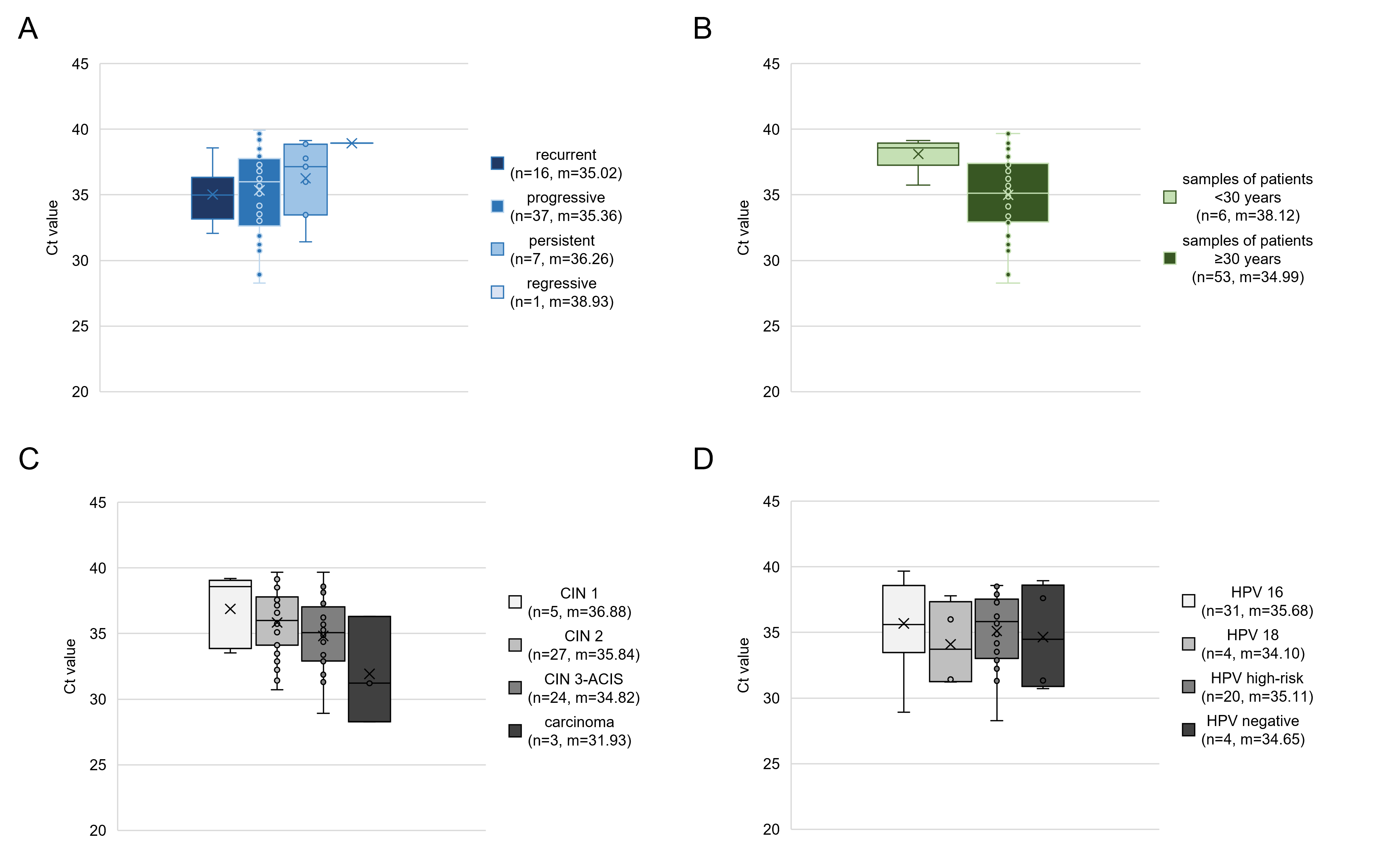

Supplement: Supplementary file 1 [file cancers-17-03095-s001.zip › Figure S1.png]
